# Multi-Omics Approach Reveals the Potential Core Vaccine Targets for the Emerging Foodborne Pathogen *Campylobacter jejuni*

**DOI:** 10.3389/fmicb.2021.665858

**Published:** 2021-06-24

**Authors:** Hengchun Cao, Hanxiao Xu, Chunhui Ning, Li Xiang, Qiufang Ren, Tiantian Zhang, Yusen Zhang, Rui Gao

**Affiliations:** ^1^School of Mathematics and Statistics, Shandong University, Weihai, China; ^2^School of Control Science and Engineering, Shandong University, Jinan, China

**Keywords:** *Campylobacter jejuni*, vaccine, pangenome, virulence, multi-omics

## Abstract

*Campylobacter jejuni* is a leading cause of bacterial gastroenteritis in humans around the world. The emergence of bacterial resistance is becoming more serious; therefore, development of new vaccines is considered to be an alternative strategy against drug-resistant pathogen. In this study, we investigated the pangenome of 173 *C. jejuni* strains and analyzed the phylogenesis and the virulence factor genes. In order to acquire a high-quality pangenome, genomic relatedness was firstly performed with average nucleotide identity (ANI) analyses, and an open pangenome of 8,041 gene families was obtained with the correct taxonomy genomes. Subsequently, the virulence property of the core genome was analyzed and 145 core virulence factor (VF) genes were obtained. Upon functional genomics and immunological analyses, five core VF proteins with high antigenicity were selected as potential core vaccine targets for humans. Furthermore, functional annotations indicated that these proteins are involved in important molecular functions and biological processes, such as adhesion, regulation, and secretion. In addition, transcriptome analysis in human cells and pig intestinal loop proved that these vaccine target genes are important in the virulence of *C. jejuni* in different hosts. Comprehensive pangenome and relevant animal experiments will facilitate discovering the potential core vaccine targets with improved efficiency in reverse vaccinology. Likewise, this study provided some insights into the genetic polymorphism and phylogeny of *C. jejuni* and discovered potential vaccine candidates for humans. Prospective development of new vaccines using the targets will be an alternative to the use of antibiotics and prevent the development of multidrug-resistant *C. jejuni* in humans and even other animals.

## Introduction

*Campylobacter jejuni* is the most common bacterial cause of foodborne diarrheal disease worldwide, and the incidence of campylobacteriosis in humans has increased in recent decades (Young et al., 2007; Burnham and Hendrixson, 2018). As *C. jejuni* has a broad host range, it causes high morbidity in humans and in some avian species and results in huge economic losses in both developed and developing countries (Kaakoush et al., 2015; Geissler et al., 2017; Schnee and Petri, 2017; Crofts et al., 2018). In addition, *C. jejuni* infection is one of the most common risk factors for developing Guillain–Barre syndrome (GBS) that leads to nerve damage, muscle paralysis, and sometimes death (Willison et al., 2016; St Charles et al., 2017; Wijdicks and Klein, 2017).

In recent years, the overuse and misuse of antibiotics in human medicine and animal husbandry have led to an escalation of antibiotic-resistant infections (Iovine, 2013; Lopes et al., 2019; Makabenta et al., 2020). This situation is more serious for *C. jejuni* because it is likely to undergo spontaneous gene mutations as well as to acquire the antibiotic-resistant genes from the environment or other organisms (Beauchamp et al., 2017). The World Health Organization (WHO) has listed *Campylobacter* spp. as a serious threat to global public health (Silva et al., 2011). Gastroenteritis caused by *C. jejuni* is clinically similar to other bacteria and is usually treated empirically with fluoroquinolones and tetracyclines (Iovine, 2013; Sierra-Arguello et al., 2018; Efimochkina et al., 2020). To address the huge threat brought by antibiotic-resistant strains of *C. jejuni*, alternative intervention strategies are presently sought. New vaccine development is one of the alternative strategies to effectively prevent the infection and avoid the use of antibiotics (Astronomo and Burton, 2010; Hill et al., 2016). Compared with other foodborne bacteria, the development of vaccines against *C. jejuni* is more challenging due to its antigenic diversity, insufficient understanding of its pathogenesis, and the lack of experimental animal models to elucidate the development of disease and test the potential vaccine candidates (Riddle and Guerry, 2016). To date, attempts to develop a clinical vaccine have been futile in humans, and more research is needed into the development of *C. jejuni* vaccines (Poly et al., 2019).

Unlike other enteric bacterial pathogens, *C. jejuni* lacks or does not rely extensively on many classical virulence factors (Burnham and Hendrixson, 2018). For example, the flagella of *C. jejuni* promote the anti-inflammatory axis through the siglec-10 receptor, whereas the other gastrointestinal pathogens activate the TLR5 receptor (Stephenson et al., 2014). Elucidation of the mechanism of pathogenesis and, thus, vaccine development faces additional challenges due to its unique characteristics. Pangenome analysis provides a framework for exploring the genomic diversity in one species and helps identify new candidates for vaccine development and a new generation of antimicrobials (Croucher et al., 2017; Alam et al., 2020). *C. jejuni* is known to be a highly diverse species and displays extensive genetic variations. Therefore, analyzing the core genome shared by all strains may be an appropriate strategy to identify the possible conserved molecular determinants of virulence factors (VFs) for use as vaccine targets. At present, reverse vaccinology, the process of antigen discovery from genome information and vaccine development, has been applied in generating vaccines for many other pathogens (Hassan et al., 2016).

In this study, all available complete genome sequences of the *C. jejuni* strains in the National Center for Biotechnology Information (NCBI) database have been analyzed and its pangenome has been estimated. The average nucleotide identity (ANI) value was first calculated and the filtered genome sequences were applied for further analysis. Identification of the bacterial virulent proteins can benefit the understanding of the mechanisms of virulence and the design of drugs and vaccines. Because of the high genome plasticity and the diverse antigenicity of *C. jejuni*, the core VF genes have been selected as potential vaccine candidates, which will facilitate covering the entire species. After a series of analyses such as essentiality, non-host homologs, molecular weight, and cellular location, the candidate core VF proteins were screened out. To further explore the virulence of these candidate proteins, we also analyzed some experimental data in several model animals including human and pigs. The core VF targets found in this study have been shown to have high virulence from these experiments. Furthermore, immunological, functional genomics, and protein–protein interaction (PPI) analyses were performed to detect the antigenicity, molecular function, and biological processes of the potential core vaccine targets. The study highlighted the pangenomics and sequence-based reverse vaccinology in identifying potential vaccine targets for human, and the multi-omics approach will facilitate discovering the unique vaccine candidates and prevent challenging bacterial infections.

## Materials and Methods

### *C. jejuni* Genome Sequencing Data and Annotation

In order to have a comprehensive overview of the species of *C. jejuni*, the study included all available complete genome sequences (including the complete and chromosome-level sequences) of *C. jejuni* strains from NCBI^1^. The drafts and incomplete genomes were excluded in order to be standardized in the analysis. To ensure the consistency and reliability of annotation and gene prediction, the program Prokka was used to obtain the protein and nucleotide sequences of each strain (Seemann, 2014). The genome sequences used in this study are summarized in Supplementary Table 1.

### ANI Analysis

In order to establish an accurate pangenome and determine the correct classification, ANI analysis of *C. jejuni* strains was performed using the python program “pyani” (v0.2.7) (Pritchard et al., 2015). The analysis was performed based on the BLAST algorithm on a local computer with default settings (Altschul, 2012). The heatmap software package (v1.0.12) in R was used to deliver the graphical heatmap and dendrogram for the ANI values.

### Pangenome Analysis

The pipeline Roary was used to perform pangenome analysis using the *gff3* files obtained from Prokka (Page et al., 2015). The determination and the selection of the core gene thresholds are critical to the accuracy of pangenome analysis. Considering the sequence data were complete and chromosome genome assemblies were of high quality, a core threshold (99% ≤ strains ≤ 100%) was used instead of a soft-core threshold (95% ≤ strains ≤ 99%), which means that the core genes could be shared by all of the *C. jejuni* strains used in this study. The expansiveness of the pangenome was estimated in R with the “micropan” package according to the Heaps’ law model (Snipen and Liland, 2015). The decay parameter alpha was calculated with 5,000 random permutations. Besides, all of the three types of pangenomes were searched to compare the distribution of their functional categories using the Clusters of Orthologous Genes (COG) database (Tatusov et al., 2000). Pathway enrichment analysis with the Gene ontology (GO) and Kyoto Encyclopedia of Genes and Genomes (KEGG) databases was performed using the “ClueGO” plugin from Cytoscape 3.8.0 (Shannon et al., 2003; Bindea et al., 2009).

### Core VF Analysis

With the aim to obtain insights into the virulence and pathogenic potential of *C. jejuni*, the VFs present in the core genome were explored using the Virulence Factors Database (VFDB) (Liu et al., 2019). For the core genome sequences, the search was performed using the program BLASTX 2.6.0+, which uses nucleotide vs. protein alignments based on a punctuation matrix (BLOSUM62). The blast search was carried out against all the virulence-associated proteins adhering to the following parameters: bit score > 100, *e*-value < 1.0e–5, and percentage identity > 30%.

### Characterization and Antigenicity Analysis of Core VFs to Be Used as Vaccine Candidates

In order to elucidate the effectiveness of potential core vaccine candidates, the core VF genes were first detected using the Database of Essential Genes (DEG) adhering to the following parameters: *e*-value cutoff = 1e–5 and minimum bit score = 100 (Zhang and Lin, 2008). The core VF proteins of *C. jejuni* were aligned with human protein sequences to search for the human homologues, thereby eliminating the chance of autoimmunity. These proteins with percent identity < 35% and *e*-value < 0.005 were considered as non-host bacterial proteins. In order to facilitate purification, cloning, and expression, a series of analyses was carried out to characterize the filtered proteins. The prediction of transmembrane helices plays an important role in the study of membrane proteins. TMHMM is a widely used bioinformatics tool based on the hidden Markov model (HMM) and is usually used to predict the transmembrane helices of integral membrane proteins. Thus, the software TMHMM v2.0 was used to predict the transmembrane helices, and the proteins with less than two transmembrane helices were selected. PSORTb v3.0 was applied to predict the most probable localization (Yu et al., 2010). The molecular weights of the prioritized genes were estimated by using Expasy PI/MW tool^2^. Proteins with molecular weights < 110 kDa were eventually designated as potential vaccine candidates because they are easy to purify and can be effectively used for vaccine development. In addition, VaxiJen v2.0 was used to check the antigenicity of the filtered proteins with a threshold value greater than 0.4 (Doytchinova and Flower, 2007; Zaharieva et al., 2019). The BCPreds server 1.0^3^ was used to identify B cell epitopes with a default epitope length of 20 and a specificity of 80% using the method by BCPreds. IEDB analysis resource^4^ was used to predict the T cell epitope peptide [major histocompatibility complex (MHC) I and MHC II] with the method of “IEDB recommended 2020.09” (Kim et al., 2012). In the end, the proteins that are virulent, essential, non-host homologous, exposed or secreted with less than two transmembrane helices, and with high antigenicity were chosen as candidate proteins.

### Three-Dimensional Structure and PPI Analyses of the Potential Vaccine Candidates

We used the Protein Data Bank and SWISS-MODEL to explore the three-dimensional (3D) structures of the query proteins through comparative modeling (Sussman et al., 2010; Marco et al., 2014). In order to predict the PPIs, the STRING database was used for the candidate proteins (Szklarczyk et al., 2011). The interaction score of these functional proteins was chosen to be 0.8, which means that the connections between the nodes and lines have a high degree of credibility. PPI analysis of the candidate proteins benefits the understanding of the molecular mechanisms and biological processes and for determining the potential therapeutic targets.

### Transcriptomic Analysis

To test the virulence of the potential core vaccine targets, additional transcriptome analysis was performed using previous RNA sequencing (RNA-seq) data of *C. jejuni*. The raw data were downloaded from the BioProject PRJNA473070 and PRJNA615847 of the European Nucleotide Archive (ENA) in the European Molecular Biology Laboratory (EMBL) database. Reads were mapped to the *C. jejuni* 81–176 and F38001 genomes using Bowtie 2 (version 2.2.9) and counted with featureCounts (version 2.0.1) (Langmead and Salzberg, 2012; Liao et al., 2014). Differential expression was analyzed using the DESeq2 package (version 1.28.0) and a volcano plot was visualized using ggplot2 (version 3.3.2) in R platform (version 4.0.3).

## Results and Discussion

### Genomic Relatedness and Characterization of *C. jejuni* Strains

**FIGURE 1:**
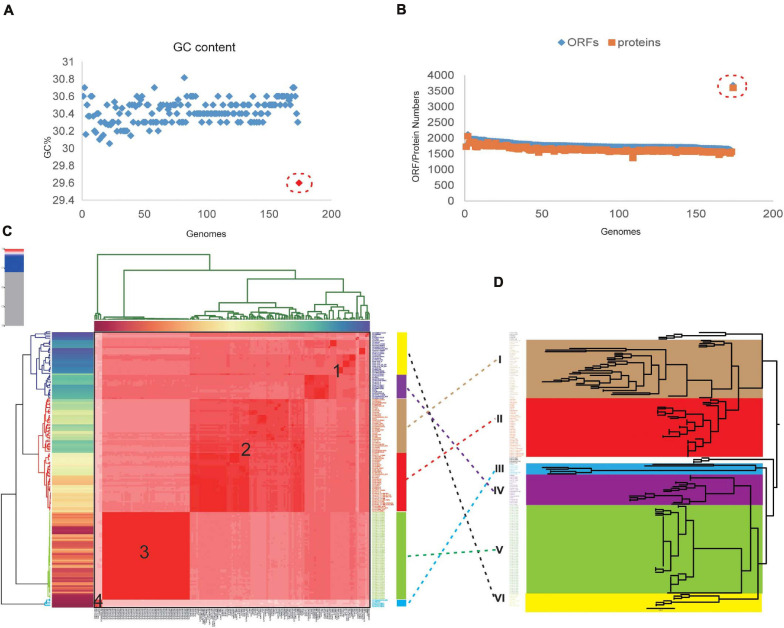
Taxonomic classification of *Campylobacter jejuni* based on comparison at the whole-genome level. **(A)** The GC content of 174 original complete genomes in NCBI. Strain 414 is highlighted with the *red circle*, which is obviously far away from the other strains. **(B)** Numbers of genes and open reading frames (ORFs) of 174 complete genomes. Strain 414 is highlighted with the *red circle*. **(C)** Phylogenetic tree based on BLAST all vs. all of the 173 *C. jejuni* strains, which could be divided into four groups (numbers *1*, *2*, *3*, *4* marked in the *red area*). The *vertical rectangular boxes* correspond to the relevant clades in the core genome tree (the *different colors of the boxes* match the clades on the *right*, e.g., the *red box* corresponds to clade III). **(D)** Core genome tree based on the 911 core genes of 173 strains. *Different colors* represent the diverse clades.

In this study, a total of 174 complete genome sequences of *C. jejuni* strains collected from different geographic locations and isolation sources were preliminarily analyzed (Supplementary Table 1). To be consistent with the genomic data, all of the sequences were annotated using the software Prokka (Seemann, 2014). The correct taxonomy classification is essential for obtaining high-quality pangenomes (Wu et al., 2020). In order to determine the taxonomic status and obtain a high-quality pangenome of *C. jejuni*, the ANI values were firstly calculated to estimate the genetic relatedness among the strains. ANI has become one of the main genome options for DNA–DNA hybridization for taxonomic purposes (Arahal, 2014). The previously suggested species threshold of 95% ANI can represent the same species (Goris et al., 2007). We found that the ANI value of the *C. jejuni* strain 414 is about 91%, which is obviously different from the other 173 strains and may be an incorrect classification (Supplementary Figure 1A). Moreover, the GC content, open reading frame (ORF), and the protein numbers of strain 414 were also significantly different from the other strains (Figures 1A,B). In accordance with a previous study, we highly suspect that strain 414 may belong to *Campylobacter coli* or other *Campylobacter* subspecies (Hepworth et al., 2011; Supplementary Figure 1B). In order to improve the quality and accuracy of the whole-genome analysis of *C. jejuni*, the genome sequence of strain 414 was removed in subsequent analyses.

As expected for the remaining 173 strains belonging to the same species, higher ANI values ranging from 95.52 to 99.99% were observed and could be divided into four groups (Figure 1C). The GC content, genome size, and the number of ORFs and proteins for all of the analyzed genomes were determined (Supplementary Table 1). The lengths of the obtained *C. jejuni* genomes varied from 1,558,306 bp (strain 104) to 1,891,235 bp (strain NCTC11951), equal to an approx. 21% difference. The average ORF count was found to be 1,796, with the lowest number of ORFs of 1,647 (strain CJ090CC1332) and the highest number of ORFs of 2,113 (strain NCTC11951); the difference between them was approx. 28%. These vast differences supported the high diversity of the genome of *C. jejuni*.

### Phylogenetic Analysis at the Core Genome, Pangenome, and Whole-Genome Levels

Phylogenetic analysis was performed for all the included *C. jejuni* strains to understand the pattern of evolution. Using the whole-genome and core genome alignment concatenation approach, phylogenetic trees for the set of 173 genomes were constructed, and the constructed phylogenetic trees showed high phylogenetic diversity and clear evolutionary relationships among the strains (Figures 1C,D). Based on the topological structure and the evolutionary distances, the core genome tree could be divided into six main clades, in which nine strains were diverged independently of the other members. *C. jejuni* RM1285 is an environmental isolate from a chicken farm and exhibits a rod-shaped variant morphology. This strain has an increased resistance to inactivation by high hydrostatic pressure compared to the other *C. jejuni* strains (Gunther et al., 2015). Strains RM3420 and RM1285 both belong to serotype HS:19, which is believed to be a unique clone that is associated with GBS and gastroenteritis (Misawa et al., 2001; Parker et al., 2017). The stains ATCC33560 and FDAARGOS_295 are quality control strains used for the antimicrobial susceptibility testing of *Campylobacter* spp. (Huang et al., 2012). These strains are diverse from other strains since they originate from various sources and possess different virulent characteristics. The six clades could also be associated with the four groups based on whole-genome alignment, which indicated that there is a conservative relationship among these *C. jejuni* strains (Figure 1C). In fact, the phylogenetic groups in the ANI heatmap were connected with the core phylogenetic clades, such as clades III and V of the core genome phylogenetic tree being similar to groups 3 and 4 (Figure 1D). Because the core genome contained only conserved genes and was relatively small in this elucidation here, this might lead to different phylogenetic relationships between them. In addition, other studies have also tried to distinguish the closely related *C. jejuni* genomes based on the core or whole-genome sequencing type schemes and found that some clinical strain genomes are more stable than others for over a decade (Cody et al., 2013; Nennig et al., 2021). It is known that *C. jejuni* has a high genetic diversity with very few repeat sequences and virtually no inserted or phage-associated sequence in the genome (Parkhill et al., 2000). These different methods could benefit the exploration of the diversity of *C. jejuni* in different ways.

In order to further explore the phylogenetic relationships, a pangenome tree was also constructed, which showed high phylogenetic diversity. The pangenome tree could be divided into seven groups (Supplementary Figure 2A) and showed an obvious difference from the core phylogenetic tree. For example, clade V (green in Supplementary Figure 2B) contained 56 strains, which had the same evolutionary distance in the core and whole-genome trees. These strains were isolated from human feces and blood, which nearly belonged to the same BioProject PRJNA268846 (except strain CJ066CC508). Because the 911 core genes in those strains were conserved, this led to the equidistance in the core phylogenetic tree. Compared with a previous study, the core genome number has changed, which could be caused by the algorithms and the cutoff values, as well as the source and genomic diversity of the strains. But the core phylogenetic tree could also allow for the efficient identification of these closely isolated strains, just like the core genome multilocus sequence typing (cgMLST) scheme of *C. jejuni* (Cody et al., 2017). While in the pangenome tree, these strains (groups a–c and e–g) shared more accessory and unique genes that have been observed in the close lineages, which could lead to different virulence and evolution processes. Besides that, these strains in clade V belonged to one clonal complex (CC) of sequence type (ST) 667, which was similar to that in another study of ST-45 CC. Because both the core and pangenome could harbor polyphyletic and paraphyletic STs, this led to the different groups in the phylogenetic analysis (Llarena et al., 2016). Therefore, the pangenome phylogenetic analysis will facilitate the evolutionary analyses of species phylogeny reconstruction and trace the disease outbreak or the pathogenicity of different *C. jejuni* strains (Computational Pan-Genomics Consortium, 2018). The phylogenetic trees of the whole genome, core genome, and pangenomes showed diversity, which may be due to the changes in the genes among these strains under selective pressure during the evolution process; gene duplications and/or large amplifications and horizontal gene transfer (HGT) could have also contributed to the variations (Crofts et al., 2018). Therefore, the size of the genome and the number of genes in the population are variable and reflect the high plasticity and phylogenetic relationships of the *C. jejuni* genomes.

### Pangenome of *C. jejuni*

**FIGURE 2:**
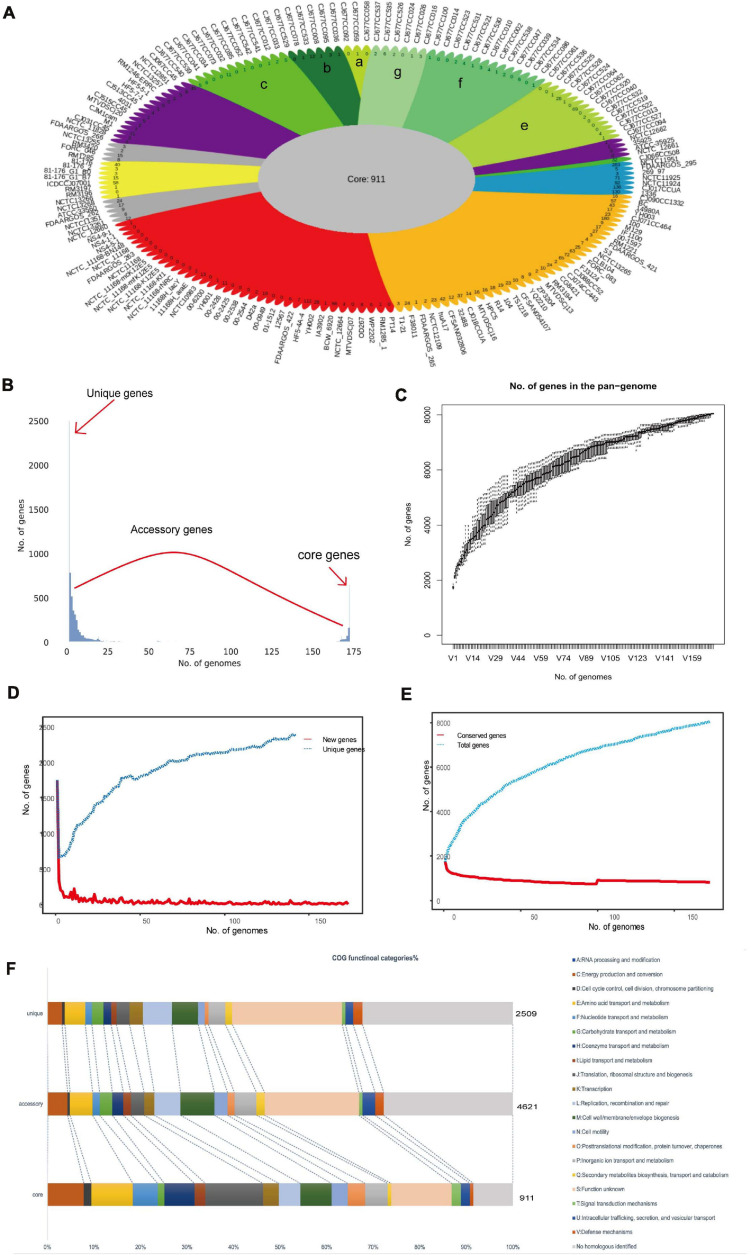
Pangenome shape of *Campylobacter jejuni*. **(A)** Pangenome flower plot showing the core genome and the different unique genes for each strain. *Different colors* represent the subgroups in the pangenome tree (the *colors* correspond to the different clades in the core genome tree). The number of subgroups is labeled with *lowercase letters*, except “*d*,” which includes the rest of the region between *two purples*. *Different shades of green* are used to distinguish the different subgroups, which correspond to clade V of the core genome tree. **(B)** Gene accumulation curves for the pangenome. Cumulative sizes were calculated by selecting strains without replacement in random order 1,000 times. **(C)** Histogram of the prevalence of the different gene families in the pangenome. A total of 8,041 non-redundant gene families identified in 173 genomes are based on their frequency distribution. Three gene categories are clearly distinguished, highlighting the unique genes (genes that only exist in one strain), core genes (gene families are present in all the analyzed strains), and accessory genes (gene families that exhibit variable frequencies). **(D)** Heaps’ law graph representation of new genes (*red line*) and unique genes in 173 genomes. **(E)** Heaps’ law graph representation of the conserved genes (*red line*) and the total genes in 173 genomes. **(F)** Distributions of the Clusters of Orthologous Genes (COG) categories in the core, accessory, and unique genes without homologs were marked in *gray*.

Pangenome analysis revealed 8,041 non-redundant genes, including 911 core genes, 4,621 accessory genes, and 2,509 unique genes (Figure 2A). Based on the Heaps’ law model, we obtained the alpha value of 0.99 and exhibited an open pangenome structure of the gene accumulation curve (Figure 2C; Tettelin et al., 2008). Due to the existence of an expanded library of the dispensable genes from various strains, the pangenome was relatively large (Figures 2B,D,E). The proportion of accessory genome (58%) was greater than that of the core genome (11%), which was also a characteristic of an open pangenome (Brockhurst et al., 2019). This situation indicates that a large and uncertain additional genome is needed to identify all the genes that are accessible to this species and ensures that *C. jejuni* responds quickly to various environments or hosts (Donati et al., 2010). COG analysis suggested that the accessory and unique genes are abundant in the binding and metabolic processes (Figure 2F). Thus, these diverse accessory and unique genes may be responsible for the high pathogenicity to humans and the potential host specificity for these strains (Gripp et al., 2011).

### Core VF Genes of *C. jejuni*

**FIGURE 3:**
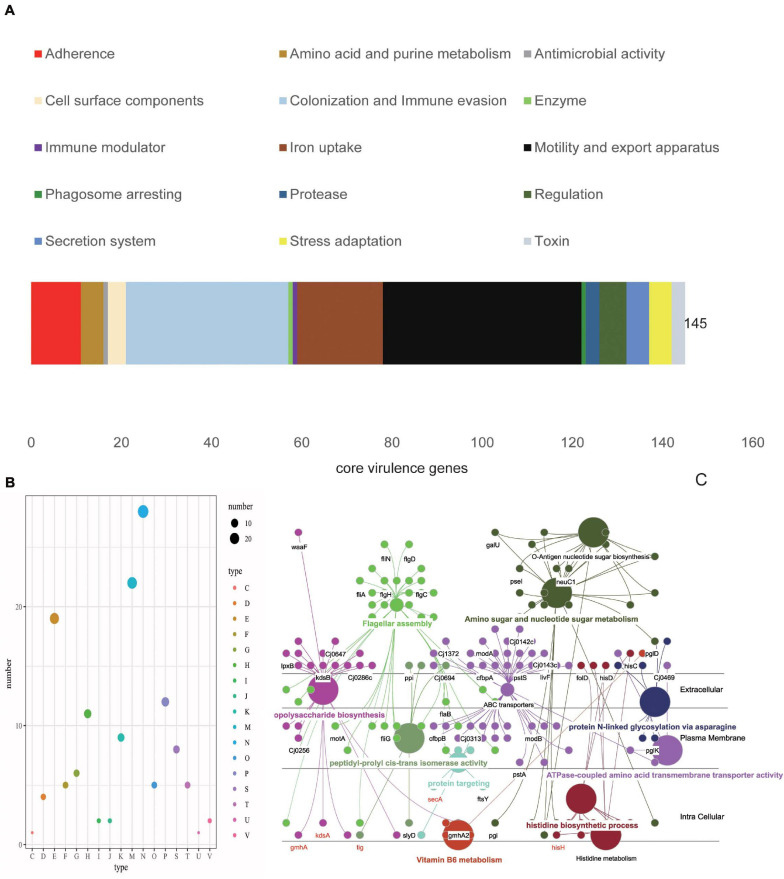
Characterization of the core virulence factors (VFs). **(A)** Distributions of the virulence type categories of 145 VF proteins. The top three types are motility and export apparatus (*black*), colonization and immune evasion (*light blue*), and iron uptake (*brown*). **(B)** Distributions of the Clusters of Orthologous Genes (COG) types of core VFs, with the largest COGs mainly distributed in cell motility (*N*) and cell component (wall, membrane, and envelop) biogenesis (*M*), which accounted for more than 35% of the 145 core VFs. **(C)** Simulated location distributions of some core VFs based on Gene Ontology (GO) functional analysis that are mainly involved in motility, biosynthesis, metabolism, and transportation.

Identification of the bacterial virulent proteins can benefit the understanding of the mechanisms of virulence and the design of drugs and vaccines. *C. jejuni* shows extensive genetic variations, and its core genome is smaller than the core genome of other foodborne pathogens (Zhou et al., 2018; Flament-Simon et al., 2020). Compared to other foodborne pathogens, very little information is known in relation to the pathogenic potential and virulence repertoire of *C. jejuni* (Young et al., 2007; Crofts et al., 2018). In order to obtain insights into the pathogenic potential, the current study explored the virulent genes in the core genome. In the end, 145 VF genes were found to be in the core genome (∼16%). In the conserved virulome of *C. jejuni*, the major VFs, including the proteins of adherence, iron uptake, motility and export apparatus, as well as colonization and immune evasion, were identified. These VFs may facilitate the pathogenesis and probable survival under adverse conditions of the organism (Figure 3A). COG category analysis showed that cell motility, membrane biogenesis, and amino transport and metabolism were the top three functions, which play important roles in pathogenesis (Figure 3B). GO functional analysis demonstrated that these core VF proteins have extensive connections with many other VFs and were mainly located in the plasma membrane, periplasmic space, outer membrane, or secreted, which means that they could be preferred as effective vaccine candidates (Figure 3C; Zagursky et al., 2003). Among the proteins, CadF is an outer membrane protein that binds to fibronectin and has been proven to be an important pathogenic factor for *Campylobacter* spp. (Talukdar et al., 2020). SodB was found to be an ingredient in vaccines against *C. jejuni* infection in broiler chickens, although antibodies to SodB-inoculated chickens detected the protein in the periplasm rather than in the membrane portion or on the surface of the bacteria (Chintoan-Uta et al., 2015). Another study found significantly higher levels of antigen-specific IgY in chickens vaccinated with glycosylated SodB, possibly due to differences in the vaccine design, *C. jejuni* challenge strains, chicken lines, injection times, adjuvants, or the vaccines themselves, such as the different glycosylation efficiencies or states of the vaccine (Alemka et al., 2013; Vohra et al., 2020). Meanwhile, SodB has been shown to be involved in oxidative stress and host cell survival, requiring further studies to increase the level and duration of protection and to facilitate the development of vaccines against *C. jejuni* in humans (Novik et al., 2010). Chemotaxis proteins like CheA, CheB, CheV, CheW, and CheY are mainly involved in the commensal and pathogenic lifestyles and have the functions of sensing, signal transduction, and signal amplification during chemotactic response (Korolik, 2019; Reuter et al., 2020). Capsular polysaccharide (CPS) and lipooligosaccharide (LOS) are very variable VFs and play critical roles in the adhesion and invasion of epithelial cells and in serum resistance and colonization (Richards et al., 2013; Islam et al., 2018; Hameed et al., 2020). A total of 37 flagella genes (∼25%) showed up in the core VF sets, which play vital roles in the rotation and switching of the bacterial flagella and are conducive to adhesion and biofilm formation (Paul et al., 2011; Burnham and Hendrixson, 2018; Cohen et al., 2020). In addition, the flagellar protein export apparatus was proven as a type III export system of *C. jejuni* and required the secretion of the *Campylobacter* invasion antigens (Cia proteins), which play roles in *C. jejuni* motility and host cell invasion (Konkel et al., 2004; Neal-McKinney and Konkel, 2012; Samuelson et al., 2013; Negretti et al., 2021). The motility, adhesion, and entry of bacteria into intestinal epithelial cells are considered to be key steps in the development of pathogenesis following exposure to *C. jejuni* (Pizarro-Cerdá and Cossart, 2006; Young et al., 2007; Henderson et al., 2020). Therefore, these core VFs can provide a new perspective on pathogenicity at the species level (Supplementary Table 2).

### Core VF Estimation for Essentiality and Non-host Homologs

Essential genes are composed of the minimum set of genes required to support cell life and have greater therapeutic potentiality (Peters et al., 2016). The essential genes in bacteria are effective therapeutic targets, especially when the bacteria confer multidrug resistance. Thus, the identification of essential genes is a key step in designing therapeutic targets for bacterial infections. Among the 145 core VFs, 94 (∼65%) were predicted as essential genes (Supplementary Table 3). These genes are mainly involved in biological processes like ATP binding, DNA binding, and transferase and permease activities. Afterward, the essential core VFs were aligned with the human proteome to confirm whether there is any similarity between them. Seventy-four proteins showed hits below the threshold value and were considered as non-host homologous proteins (Supplementary Table 4). These non-host homologous proteins can be preferably used for *C. jejuni* vaccine development to avoid autoimmune response or recombination and integration events in humans.

### Characterization and Antigenicity of Vaccine Candidate Proteins

In order to improve the efficiency, the selected proteins were characterized and their antigenicity was analyzed. Because the transmembrane and subcellular localization of proteins will affect the identification of therapeutic targets, TMHMM Server 2.0^5^ was used, and 69 candidate proteins with no transmembrane helices were finally obtained. Besides, proteins located in the periplasmic region, in outer membranes, and extracellularly are considered as effective vaccine candidates (Supplementary Table 5). Consequently, the estimation of the core VF proteins for subcellular location revealed that 47 proteins were cytoplasmic, 19 were located in the cytoplasmic membrane, one was in the outer membrane, three were unknown, and four were periplasmic (Figure 3C). It is known that outer membrane vesicles (OMVs) are a molecular complex consisting of lipopolysaccharides (LPS), outer membrane proteins, periplasmic proteins, lipids, and even cytoplasmic proteins, which are important vehicles for the simultaneous delivery of many effector molecules to host cells (Cai et al., 2018; Singh et al., 2021). Exposed proteins are often attractive targets for vaccine design, but sometimes not all proteins must be exposed to the surface, including some periplasmic proteins present in OMV preparations, which may also elicit an immunogenic response (Awanye et al., 2019). Due to the role of OMVs in intestinal adhesion and invasion, and in regulating the dynamic interaction between host and pathogens, OMVs have become potential vaccine targets for a variety of intestinal pathogens (Singh et al., 2021). Therefore, the bacterial cell surface and secreted proteins, usually located in the extracellular, periplasmic, and outer membranes, could be more effective as vaccine candidates or diagnostic targets.

Finally, five candidates of core VF proteins met the requirement and were selected as the potential vaccine targets (Table 1). Also, the alignment of these genes was well conserved among the different *C. jejuni* strains (Backert et al., 2018; Konkel et al., 2020; Moballegh Naseri et al., 2020; Vohra et al., 2020). CadF is an outer membrane fibronectin-binding protein and could be overexpressed under oxygen-enriched conditions, which means that it plays a great role in the insert surface adhesion process and promotes adhesion in oxygen-enriched conditions of *C. jejuni* (Sulaeman et al., 2012). SodB has a molecular function in metal ion (e.g., Fe) binding and superoxide dismutase activity and plays an important role in the intracellular survival of bacteria to prevent reactive oxygen species (ROS) damage. According to reports, the iron regulation system is essential for the colonization and persistence of *C. jejuni* (Ducarmon et al., 2019). HtrA has a serine-type endopeptidase activity and plays important roles in regulating the quality and quantity of protein in various bacterial infections (Backert et al., 2018). In recent years, it has been found that extracellular HtrAs can directly target the surface proteins of bacterial and host cells to promote the virulence of several pathogens, which is thought to be a new pathogenic mechanism (Skorko-Glonek et al., 2013; Heimesaat et al., 2014; Backert et al., 2018; Zarzecka et al., 2019). The extracellular HtrA protein is thought to be an ideal therapeutic target in many bacteria because of its accessibility, significance in virulence, and immunogenicity (Skórko-Glonek et al., 2017). Also, the HtrA of *C. jejuni* has been proven to be a periplasmic stress response protein that could also be secreted into an extracellular medium (Boehm et al., 2015, 2018; Neddermann and Backert, 2019). FlgC is a flagellar basal-body rod protein involved in the binding with the intestinal mucosa in poultry (Shippy et al., 2014). Under oxygen and antibiotic stresses, FlgC and several other flagellar genes were significantly upregulated and considered as components of *C. jejuni* OMVs, which participated in the virulence (Godlewska et al., 2019). KpsD is an important component of the capsular polysaccharide export system and has been predicted to be the outer membrane translocon for “group 2” capsular polysaccharides (Whitfield, 2006; Diao et al., 2017). Antigenicity analysis of the protein sequences can benefit the identification of specific antigenic peptides. It was found that all of these five proteins have high potential antigenicity and exhibit molecular weights of <110 kDa, which are conducive to purification during the experiment (Supplementary Table 6). The prioritization and filtration of the candidate proteins could help reduce the time, labor, and resources for developing potential vaccines and optimize the success rate of obtaining the best drugs or vaccines against the pathogen. These five core VFs represent attractive targets for antibacterial therapy and can serve as suitable candidates for vaccine development.

**TABLE 1:** Characterization and antigenicity of the core vaccine target proteins.

Protein name	Location	PsortB score	TMHMM prediction	Molecular weight (kDa)	VaxiJen score	VaxiJen prediction
SodB	Periplasmic	9.44	Outside	24.81	0.5003	Probable antigen
FlgC	Periplasmic	9.44	Outside	18.30	0.4831	Probable antigen
HtrA	Periplasmic	9.76	Outside	51.01	0.5379	Probable antigen
KpsD	Periplasmic	9.44	Outside	60.84	0.4261	Probable antigen
CadF	Outer membrane	10	Outside	36.00	0.8043	Probableantigen

### Structure Analysis of Core Vaccine Target Proteins

Visualization of the 3D protein structure helps in understanding the sequence patterns, functions and binding sites, as well as the interactions of the candidate proteins with the other targets. Currently, the experimentally validated 3D structure of *C. jejuni* HtrA is available in the Protein Data Bank (PDB) database (ID: 6Z05; Supplementary Figure 3A). A comparative homology model of SodB is present in SWISS-MODEL (Supplementary Figure 3B). Due to the low homology in SWISS-MODEL, the structures of the other three proteins were not predicted in the study. The cryo-electron microscopy of HtrA showed that it has two PDZ domains, which play important roles in regulating substrate binding, the proteolytic activity, and interactions between subunits. HtrA has been proven to have a close relationship in many bacteria, and the lack of functional HtrA could reduce the invasiveness of the pathogen (Skórko-Glonek et al., 2017). In addition, *C. jejuni* HtrA has the secretory function of transporting into the extracellular space and has been identified as a very attractive new vaccine target (Boehm et al., 2018). The 20-mer B cell epitope KEKSKGKNSGFQEGDIIIGV (zoomed in in Supplementary Figure 3A) is expressed on the surface of the HtrA protein and has a high antigenicity (VaxiJen value = 0.65), indicating that surface exposure and recognition of the epitope by MHC molecules could lead to a strong immunogenic response. Meanwhile, the 9-mer T cell epitope FLSLSLASA was found to have promiscuous MHC I and MHC II binding affinity and has high antigenicity scores (VaxiJen value = 0.61). These two peptides would elicit both B and T cell immune responses with a suitable adjuvant and serve as vaccine candidates. Superoxide dismutases are key enzymes in oxidative defense. SodB was predicted to have four iron sites and two manganese/iron superoxide dismutase sites in the domains of its N- (2–80 bp) and C-terminals (98–196 bp) and has high homology with the characterized 3D structure of *Helicobacter pylori* (PDB ID 3cei). Based on the sequence alignment and structural prediction, *C. jejuni* SodB might have a 20-residue C-terminal tail that might be involved in adsorption on the bacterial cell surface (Esposito et al., 2008). CadF was shown to bind fibronectin and had three cleaved forms, which could promote the binding of *C. jejuni* to polarized cells, and was thought as an important virulence determinant (Rubinchik et al., 2012; Larson et al., 2013; Konkel et al., 2020). SGGFGHYGAGVK was found in all of the three cleaved forms, EKAVEEVADTRATPQA was found in the cleaved forms CadF-1/CadF-2, and LSDSLALR of MHC II was found in both forms of CadF-2/CadF-3 (Sulaeman et al., 2012). Another *in silico* analysis of CadF also found that LSDSLALRL could be a candidate peptide for vaccine development (Moballegh Naseri et al., 2020). Thus, the epitopes SGGFGHYGAGVK, EKAVEEVADTRATPQA, LSDSLALR, and HTDNIGSRY of CadF were predicted to be suitable targets for vaccine development and required further *in vitro* studies in the next step. All of the epitope sites are summarized in Supplementary Table 7.

### PPI Analysis

PPI analysis found that these five proteins have rich connections with other proteins in the biological network (Supplementary Figure 3C). The network of HtrA also includes SodB, CadF, and several housekeeping genes, and these proteins can play great roles in the adhesion, regulation, and secretion of *C. jejuni*. For example, HrcA is a heat-inducible transcription repressor junction factor and can be transported into the extracellular space to lyse the cell. As a negative regulator of class I heat shock genes (*grpE*-*dnaK*-*dnaJ* and *groELS* operons), it can prevent the heat-induced induction of these operons. It was thought that *C. jejuni* employs a variety of protective mechanisms to resist oxidative stress as it needs to survive in an aerobic environment. This fact may result in the CadF, SodB, and HtrA proteins to be more abundant than the other membrane proteins. Also, HtrA was thought to have protease activity in different cleavage of CadF (Sulaeman et al., 2012). These interactions indicate that CadF, HtrA, and SodB may have synergistic action that could affect the adhesion, biofilm initiation, and virulence of *C. jejuni* and can serve as ideal vaccine targets for this species. CPS is a highly variable and key VF for the induction of systemic infection, and its genotype is related to GBS (Heikema et al., 2015; Sahin et al., 2017). In addition, *C. jejuni* CPS is also thought to be involved in modulating the host immune response (Maue et al., 2013). KpsD is located in the outer membrane and is an important component of CPS biosynthesis and secretion. Besides, there are several rare monosaccharide biosynthesis genes in the network, such as *hddA* and *kfiD*, which indicates that the CPS of *C. jejuni* may have abundant novel monosaccharide resources and could be the important causes of the pathogenicity (Kneidinger et al., 2002; Valvano et al., 2002; Egger et al., 2012). The flagellar-associated protein network includes more than 20 genes that are inseparable from each other. These genes are related to the motility, colonization, and chemotaxis of *C. jejuni* (Rajagopala et al., 2007; Lertsethtakarn et al., 2011; Nakamura and Minamino, 2019). PPI analysis could determine the relationships between these proteins in the network and also help in understanding the co-evolution and function of these proteins (Cong et al., 2019).

### Transcriptome Investigation and *in vitro* Verification

**FIGURE 4:**
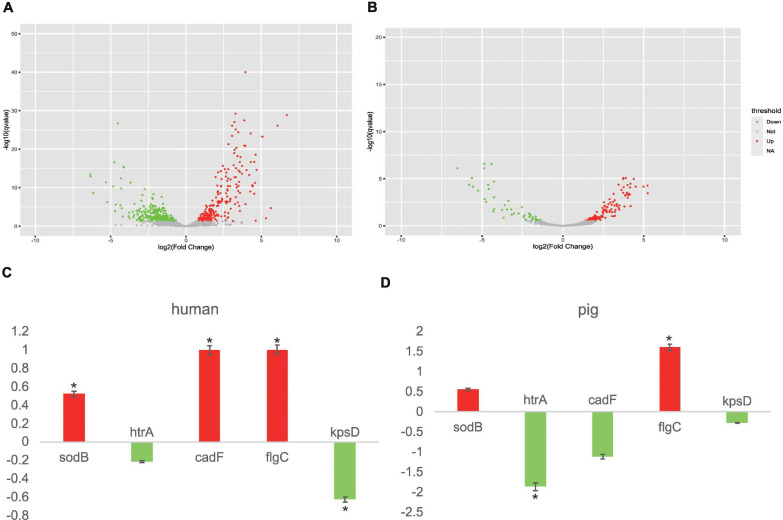
*Campylobacter jejuni* transcriptome analysis in human INT 407 and Caco-2 cells and the pig intestinal loop. **(A)** Transcript level changes between Caco-2/INT 407 and Mueller–Hinton broth. Volcano plot diagrams displaying the differentially expressed genes. Upregulated and downregulated genes are in *red* and *green dots*, respectively. **(B)** Volcano plot diagrams of the transcript level changes between the wild type (WT) and CiaD mutation in the pig intestinal loop. **(C)** Different expressions of five target genes in human. *Color* corresponds to that in **(A)**. **(D)** Different expressions of five target genes in pig. *Red* represents upregulated genes and *green* represents downregulated genes, corresponding to that in **(B)**. *P < 0.05, Fisher’s exact test.

In order to gain further insights into the responses of the potential core vaccine candidates and core VFs to different stresses, we selected two transcriptome data to analyze the gene expression in different experiments of human and pig (Negretti et al., 2019, 2020). In the environment of co-culturing with human INT 407 and Caco-2 cells, the expression levels of 126 genes, including the 25 core VFs (which include *sodB*, *cadF*, and *flgC*) that were found in this study, were increased and the expression levels of 148 genes (including 13 core VFs) were decreased under human immune stress (Figures 4A,B). For the five selected proteins, nearly all of them had an apparent differential to the stress in human and pig (Figures 4C,D). As well, the results in the pig ligated intestinal loop model showed that the expression levels of 33 core VFs, including *flgC*, have been increased and those of 23 core VFs, including *htrA*, have been decreased. The oxidative stress response genes and the iron acquisition genes, including the potential vaccine targets *htrA*, *sodB*, and other core VFs such as *chuA*, *chuB*, and *chuD*, were expected to be decreased due to the intestinal mucus in the intestinal loop of pig. This study found that the increased core VFs were mainly associated with the motility- and flagellar-related genes in both human and pigs and that the decreased core VFs were mainly related to iron transport system proteins (Supplementary Table 8). These results indicate that the flagellar genes are important VFs, which are essential for *C. jejuni* motility and the secretion of virulence proteins. The differences in the gene expressions could be caused by the different transcriptional responses by different hosts or the need for a certain reaction time after infection (Negretti et al., 2020). In this study, transcriptome analysis of *C. jejuni* indicated that the core VFs play important roles under different stresses. The candidate proteins found in this study may be efficient vaccine targets both in human and other animals. With the development of more animal models, these core VFs can provide abundant gene resources, which may be beneficial to the study of the virulence mechanisms of *C. jejuni*.

Although no clinical vaccines have been developed, several relevant animal experiments of the potential core VFs have been carried out previously. The chitosan–DNA vaccines based on CadF have induced significant protective immunity in a mouse model (Zheng et al., 2007). SodB was found to be useful as a constituent of vaccines for the control of *C. jejuni* infection in broiler birds; however, modest protection was observed late relative to the life of broiler birds, and further studies are required to potentiate the magnitude and timing of protection (Chintoan-Uta et al., 2015). Besides, the effects of serine protease HtrA on apoptosis, intestinal immune response, and extraintestinal pathology in infant mice infected with *C. jejuni* have been explored (Heimesaat et al., 2014). These experiments will help carry out further vaccine research and accelerate the development of clinical vaccines for *C. jejuni*.

## Conclusion

With the growing problem of the antimicrobial resistance of *C. jejuni*, it is an urgent need to develop and use vaccines in order to control this infectious disease. The genome of *C. jejuni* is highly plastic, and the expression patterns of the virulence genes in animals and humans are still unclear; these challenges bring great difficulties to vaccine development at present. In this study, we first examined and established the correct taxonomy classification based on ANI and phylogenetic relationship analyses. Upon comparative genomics analysis of 173 complete genomes, *C. jejuni* exhibited an open pangenome with 8,041 gene families, which could help this pathogen to adapt to diverse hosts and environments. Core VF genes are suitable candidates to develop broad-spectrum antibiotics or vaccines that could target the entire bacterial species. From the functional genomics and immunological analyses, CadF, KpsD, FlgC, SodB, and HtrA have been validated to be used as the core vaccine candidates against *C. jejuni*. The five candidates have also been proven to be effective through *in vitro* experiments, but more experiments are still needed to validate them in the future. In the case of *C. jejuni*, other proteins such as JplA, FlpA, and FlaC have been identified as candidate vaccine targets, and *in vivo* tests have been conducted in several studies (Jin et al., 2001; Baqar et al., 2008; Flanagan et al., 2009; Neal-McKinney et al., 2014). Because the proteins do not cover all strains (FlpA covers 148 strains in this study) or might be filtered by the DEG (JplA is a core VF, but not an essential protein here) or other databases, they should not be ignored, especially the other core VF proteins listed in Supplementary Table 2. Therefore, these core VFs can be used as potential effective antigens for vaccine development and can serve as alternatives to the use of antibiotics to control *C. jejuni* infection. The integrated multi-omics approach to vaccine development strategy will also be applicable to other infectious bacteria.

## Data Availability Statement

Publicly available datasets were analyzed in this study. This data can be found in the article/Supplementary Material.

## Author Contributions

YZ, RG, and HC conceived the project and prepared the manuscript. HC, HX, CN, and LX performed the sequences and other data analysis. HC, QR, and TZ conducted the bioinformatics analyses. All authors read and approved the final manuscript.

## Conflict of Interest

The authors declare that the research was conducted in the absence of any commercial or financial relationships that could be construed as a potential conflict of interest.

## Footnotes

1 https://www.ncbi.nlm.nih.gov/genome/

2 https://web.expasy.org/compute_pi/

3 http://ailab.ist.psu.edu/bcpred/

4 http://tools.iedb.org

5 http://www.cbs.dtu.dk/services/TMHMM/
