# Supplementary figures and images for: Multi-Omics Approach Reveals the Potential Core Vaccine Targets for the Emerging Foodborne Pathogen Campylobacter jejuni
Source: Front Microbiol. 2021 Jun 24;12:665858. doi: 10.3389/fmicb.2021.665858 (PMC8265506; doi:10.3389/fmicb.2021.665858)

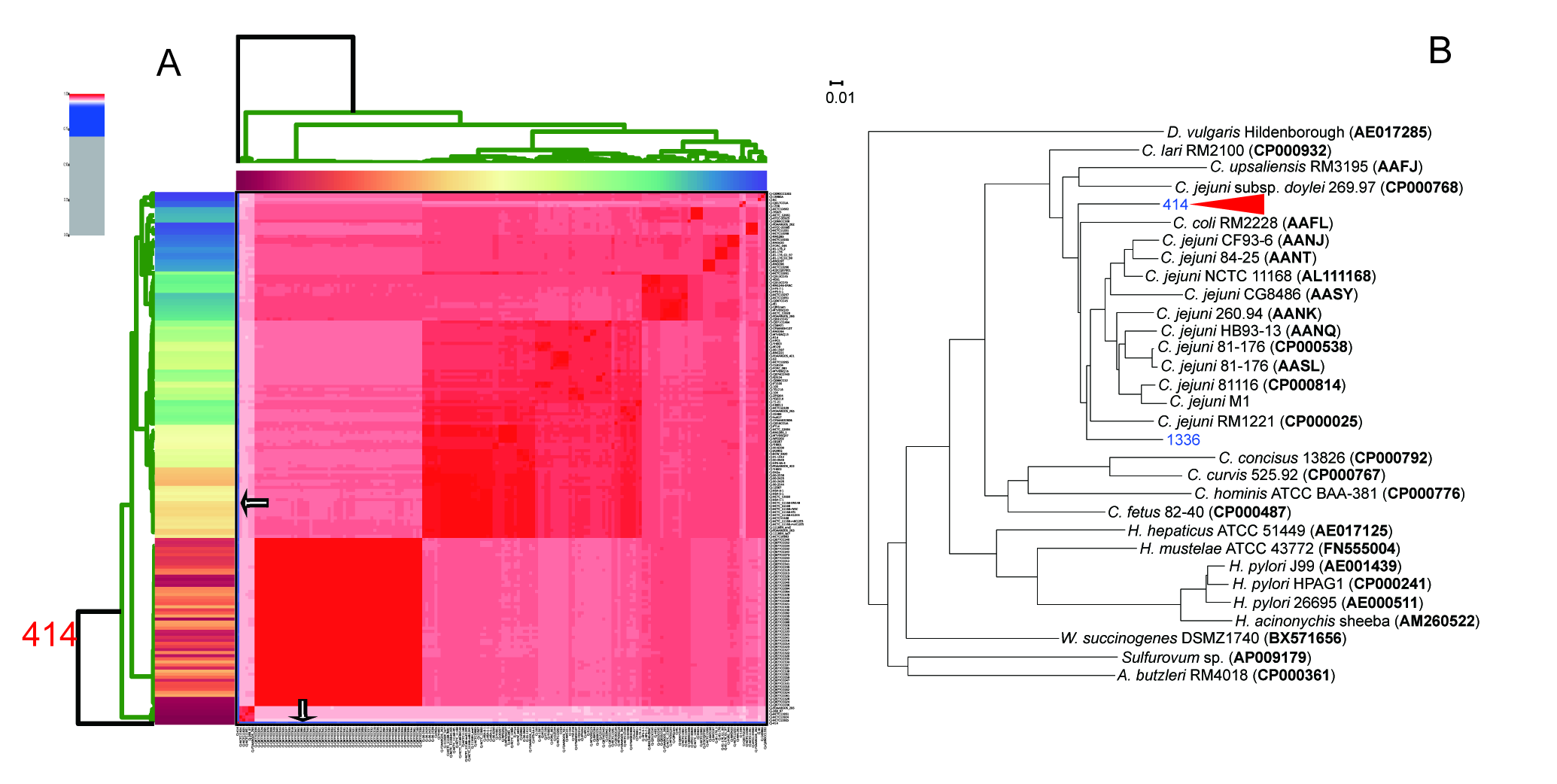

Supplement: Supplementary file 9 [file Image_1.TIF]

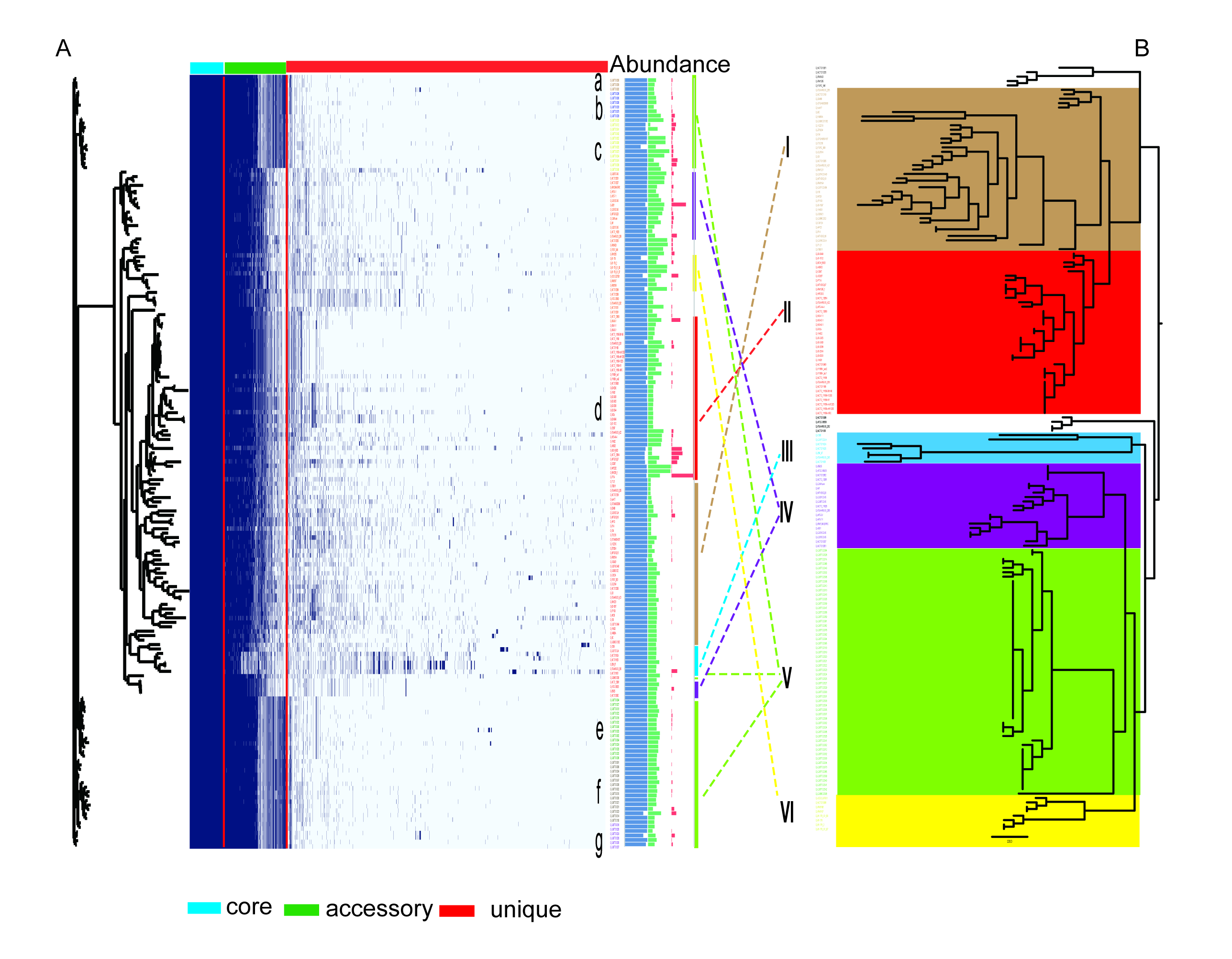

Supplement: Supplementary file 10 [file Image_2.TIF]

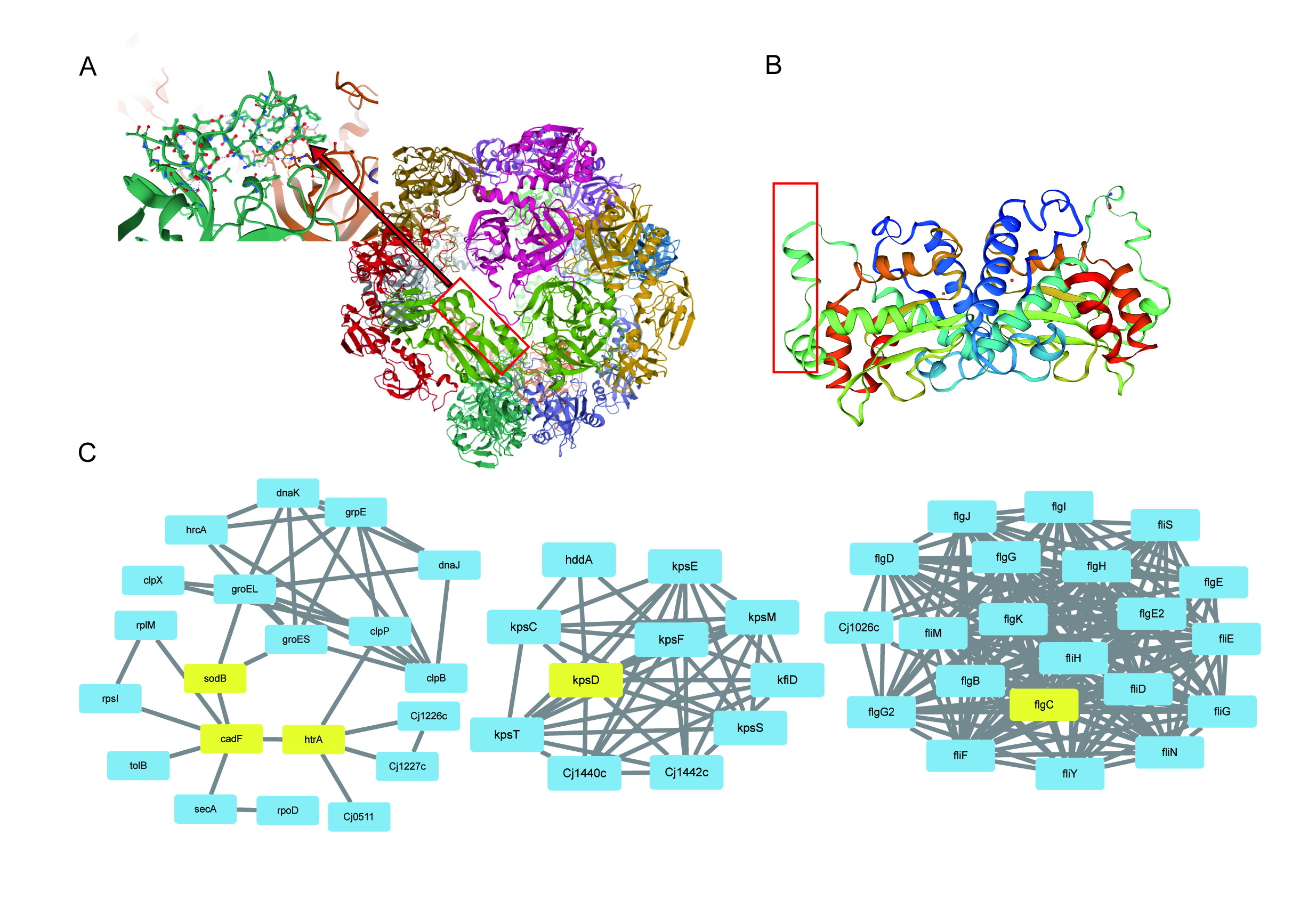

Supplement: Supplementary file 11 [file Image_3.TIF]
